# Anthropometric profiles of 8–11 year old children from a low-income setting in South Africa

**DOI:** 10.1186/s12889-019-6530-x

**Published:** 2019-03-18

**Authors:** Sandra S. Pretorius, Natalia Neophytou, Estelle D. Watson

**Affiliations:** 1 Centre for Exercise Science and Sports Medicine, School of Therapeutic Sciences, Faculty of Health Sciences, WITS Medical School, University of the Witwatersrand, Parktown, Johannesburg, 2050 South Africa; 2 INMED SA Partnerships for Children, Northriding, Johannesburg, 2169 South Africa

**Keywords:** Malnutrition, Overweight, Obesity, Urbanisation, Childhood, Anthropometry

## Abstract

**Background:**

Communities in low-to-middle income countries undergoing rapid urbanisation as well as nutrition transition are particularly at risk for associated health issues such as overweight and obesity. South Africa has a double-burden of both under- and over-nutrition, creating some uncertainty as to where to direct healthcare interventions. Therefore, providing anthropometric data in vulnerable populations, such as in early childhood, is much needed.

**Methods:**

This observational study assessed height, weight, head circumference and age-adjusted BMI in 1785 Grade 4 (9.5 ± 0.7 years old) learners from 12 schools in two different areas in South Africa.

**Results:**

Overall, the results of this study found a higher prevalence of overnutrition (>95th percentile for age-adjusted BMI) than undernutrition (<15th percentile for age-adjusted BMI), 27.3% versus 12.4% respectively. Although the boys were significantly older and taller than the girls in this sample, there were no significant sex differences between boys and girls for BMI (19.9 kg/m^2^ ± 6.0 vs 20 kg/m^2^ ± 5.8, *p* = 0.59). Significant differences were found between peri-urban and urban areas for undernutrition (16.1% versus 9.5%, *p* < 0.001) and overnutrition (9.7% versus 41.2%, *p* < 0.001).

**Conclusion:**

South African children living in urban areas are particularly vulnerable to high rates of overweight and obesity. Therefore, interventions that are area and context specific are needed to address the issues of malnutrition in South Africa.

## Background

In low-to-middle income countries (LMIC), the nutritional status of the population is somewhat complex. A pattern of malnutrition has been described, which is characterized by undernutrition in children, and high obesity rates in both adults and children [1]. In South Africa, this is particularly true, yet it has been found that over-nutrition is generally the dominant malnutrition pattern, as well as the fact that different demographic areas are affected differently by overweight and obesity [2, 3]. Contributing to this is urbanization, improved socioeconomic status (SES) and a more sedentary lifestyle [3]. According to the World Health Organization, overweight and obesity are becoming more prevalent in LMICs, particularly in urbanized areas [4]. It has been found that in African communities, increased urbanization is associated with an increased rate of obesity as well a poorer overall diet [5–7]. Since urbanization is on the increase among the African population [2], obesity rates will likely continue to increase in the future, which is of great concern.

When combining overweight and obesity prevalence for South African children aged 6–14 years, the South African National Health and Nutrition Examination Survey (SANHANES-2012) showed a prevalence of 13.5% in school children. This was higher than the global prevalence of 10%, but lower than the 32.6% prevalence of children aged 6–11 years in the USA [8, 9]. In the age group 10–14 years, the prevalence of overweight and obesity was much higher in South African girls (16.7 and 5.6%, respectively) than in boys (7.5 and 2.7% respectively) [3].

Overweight and obesity negatively affects health outcomes in children in the short- as well as long-term [3]. It can lead to asthma and a predisposition to cardiovascular disease, insulin resistance, type 2 diabetes, respiratory morbidity, impaired glucose tolerance, impaired psychological and social function and musculoskeletal discomfort [10]. Overweight and obese children also tend to be more physically inactive as compared to non-obese children, with the resultant lower expenditure of energy and therefore, places them at even greater risk of obesity and developing non-communicable diseases (NCD’s) later in life [3].

Of great concern is that the majority of children between the ages of 10 and 14 years indicated that they were happy with their current weight, even though 76.5% of them perceived themselves to have a ‘fat’ body image, while only 21.9% perceived they had a normal body image and 1.6% of them perceived they had a very thin body image. This can lead to problems when managing overweight and obesity in African populations, since they often have an inaccurate body weight perception, or are not concerned about their weight from a social or health perspective [2]. Furthermore traditional and cultural perceptions regarding body weight are often contrary to Western “healthy norms”. For instance, in African communities being overweight may represent affluence, happiness or success as well as health (not having the human immunodeficiency virus/AIDS) [11].

High obesity rates in South Africans may occur for two reasons. Firstly, there has been a change in nutrition patterns whereby typical traditional diets where fat intake only constituted 16% of the total calories consumed (noted in the 1940s), have since been replaced by diets consisting of more than 26% fat [2, 12]. Urbanized South Africans tend to follow, or are slowly shifting towards, a more Westernized diet, which has resulted in an increased consumption of saturated fats, trans-fatty acids, refined starches and added sugars [13], while their rural counterparts generally consume a diet which is high in starch-rich staple foods and dietary fiber, higher in plant protein sources (such as legumes) and lower in fat [13]. Secondly, obesity levels have increased due to urbanization which has resulted in unhealthy food becoming inexpensive as well as a reduction in energy expenditure due to a culture of labour-saving mechanical devices that reduces overall physical activity levels [1, 2, 14].

The other well recognized malnutrition pattern seen in South Africa is undernutrition. Short term effects of undernutrition include an increased risk of mortality and morbidity as well as an increase in disability; while long term effects include reduced adult size, increased mortality rates as well as impaired intellectual ability [15]. Undernutrition may also result in stunting, specifically within the first 2 years of life. Stunting may result in shorter adult stature, reduced school achievements as well as decreased birthweight in progeny [16].

Therefore, profiling the anthropometric status of children in LMICs is essential to understand the prevalence of both under- and over nutrition, in a timeous manner, which allows for potential intervention before reaching adulthood. Anthropometric measures are the most cost effective and non-invasive means for assessing growth, which in turn provide insight into child health. Since South Africa has a complex nutritional status which is ever-evolving, anthropometric information needs to be readily gathered to enable researchers to effectively monitor and intervene (if necessary) in at risk populations. This study provides anthropometric information of South African Grade 4 learners and contributes to building a clearer picture of regional overweight and obesity data in primary school children in South Africa.

## Methods

### Study setting

This observational study was part of a baseline data collection stage of a larger *Health in Action Programme*, which is being run by INMED SA [17], and aims to monitor and evaluate programme progress and impact. The *Health in Action Programme* is run in partnership with South Africa’s Eastern Cape and Gauteng Departments of Education, and aims to reach over 100,000 school children ages 6 through to 12 years old in over 100 quintile 1–3 primary schools situated in low-income communities in the Port Elizabeth (Eastern Cape province) and Johannesburg (Gauteng province) areas over a four-year period. Quintile 1–3 schools represent schools from low socio-economic areas, as classified by the National Poverty Table and determined by income, dependent ratios and literacy levels [18, 19]. The Eastern Cape province represents a more peri-urban setting whilst the Gauteng province represents a large urban area surrounding Johannesburg (one of the largest cities in Africa).

### Sample size

For the purposes of this study, only Grade 4 school children were included into the sample. With a beneficiary population size of approximately 100,000 primary school children, and with a desired 95% confidence level and 5% margin of error, a sample size of at least 370 was required. A sample size of 10% of the 116 primary schools that were expected to participate in the programme was convenience sampled, including five schools in Gauteng Province (two to three schools in each of two Johannesburg districts) and seven schools in the Eastern Cape Province (two in each of the three primary Port Elizabeth communities and one farm school). Data was collected from the selected schools at baseline at the start of the program (prior to intervention activities) during March 2016.

### Data collection procedures

All anthropometric measurements were completed by trained research assistants from INMED SA and the study team. Weight was measured with a calibrated SECA 767 electronic scale (Seca Scale Corp., Munich, Germany) and height was measured with a SECA 220 telescopic measuring rod according to acceptable standardised methods (Seca Scale Corp., Munich, Germany). The learners removed shoes, heavy outer clothing (jacket, vest, sweater, hat), and emptied all pockets (cell phones, iPods etc). To determine the height of the learners, all possible shoes, hats, and hair ornaments/buns/braids were removed, and participants stood on an uncarpeted floor with their back against the stadiometer. Participants were weighed and measured without shoes, but with clothes on, as described above, to protect their privacy, as measurements were taken in a classroom setting. Head circumference was measured using a measuring tape that cannot be stretched and the tape wrapped securely around the widest possible circumference of the head, the broadest part of the forehead, above the eyebrow and above the ears over the most prominent part of the back of the head. The measurements were taken three times and the largest measurement to the nearest 0.1 cm selected. To determine whether an individual’s weight was appropriate for height, the Quetelet index (W/H^2^) or the BMI was used and classified according to the World Health Organization’s (WHO) category’s for age- and sex- adjusted percentiles [20].

### Data analysis

Data was analyzed using STATA Version 12 (StataCorp, LP). All data were tested for normality using the Shapiro-Wilk test. Non-normal data was log transformed. For normally distributed data (weight), independent t-tests were done. For data that was still non-normally distributed after log transformation (age, head circumference, height and BMI), Mann-Whitney U tests were done. A Chi-squared test was done to determine the differences between the sexes and schools for each BMI category.

## Results

There was a total sample of *n* = 1818 grade 4 learners. Due to the effects of puberty on body composition, all learners that were 12–14 years old were removed from the analysis (*n* = 33). The anthropometric profiles of all the remaining learners (*n* = 1785), is shown in Table 1. Of the total sample, 51% were boys and 49% were girls. The average age of the learners was 9.5 years old and the average body mass index (BMI) was 19.9 kg/m^2^.

**Table 1:** Anthropometric details of Grade 4 learners (*n* = 1785)

	Mean (±SD) n (%)
Sex	
Boys	918 (51.4)
Girls	867 (48.6)
Age (years)	9.5 (±0.7)
Head circumference (cm)	48.7 (±9.2)
Height (cm)	132.8 (±6.7)
Weight (kg)	34.8 (±10.6)
BMI (kg/m^2^)	19.9 (±5.9)
BMI Categories	
< 3rd percentile	65 (3.6)
3rd-15th percentile	157 (8.8)
15th -50th percentile	503 (28.2)
50th – 85th percentile	573 (32.1)
> 95th percentile	487 (27.3)

Significant sex differences (Table 2) were found within the sample of learners, with the boys being on average 0.2 years older, with a statistically significantly larger head circumference and height. No differences were found between the boys and girls for weight and BMI, or within the BMI categories.

**Table 2:** Gender differences in anthropometric profiles

	Girls (n = 867)	Boys (n = 918)	*p*-value
	Mean (±SD) n (%)	Mean (±SD) n (%)	
Age (years)	9.4 (±0.7)	9.6 (±0.7)	***< 0.001***
Head circumference (cm)	48.4 (±9.3)	48.9 (±9.2)	***< 0.001***
Height (cm)	132.4 (±6.7)	133.2 (±6.7)	***0.001***
Weight (kg)	34.8 (±10.7)	34.8 (±10.5)	0.67
BMI (kg/m^2^)	20.0 (±5.8)	19.9 (±6.0)	0.59
BMI Categories			0.71
< 3rd percentile	28 (3.2)	37 (4.0)	
3rd-15th percentile	81 (9.3)	76 (8.3)	
15th -50th percentile	236 (27.2)	267 (29.1)	
50th – 85th percentile	284 (32.8)	289 (31.5)	
> 95th percentile	238 (27.5)	249 (27.1)	

Statistical significance set at *p*<0.05

Learners from the Port Elizabeth area were significantly older, and had a larger head circumference than their Johannesburg counterparts (Table 3). Learners from the Johannesburg area weighed significantly more, and had a higher BMI than those from the Port Elizabeth area. There were significantly more learners that were below the 15th percentile for BMI in Port Elizabeth (16.1%) versus Johannesburg (9.5%). On the other hand, Johannesburg schools had a significantly higher number of participants that were above the 95th percentile for BMI (41.2% versus 9.7%, *p* < 0.001).

**Table 3:** Geographical differences in anthropometric profiles

	Port Elizabeth Area (*n* = 791)	Gauteng Area (*n* = 994)	*p*-value
	Mean (±SD) n (%)	Mean (±SD) n(%)	
Gender			
Girls	392 (49.6)	475 (47.8)	
Boys	399 (50.4)	519 (52.2)	
Age (years)	9.7 (±0.8)	9.4 (±0.7)	***< 0.001***
8 year olds	23 (2.9)	58 (5.8)	
9 year olds	338 (42.7)	566 (56.9)	
10 year olds	322 (40.7)	321 (32.3)	
11 year olds	108 (13.7)	49 (4.9)	
Head circumference (cm)	52.7 (±2.1)	45.4 (±11.2)	***< 0.001***
Height (cm)	132.7 (±7.1)	132.8 (6.4)	0.42
Weight (kg)	31.1 (±7.2)	37.7 (±11.9)	***< 0.001***
BMI Categories			***< 0.001***
< 3rd percentile	38 (4.8)	27 (2.7)	
3rd-15th percentile	89 (11.3)	68 (6.8)	
15th -50th percentile	271 (34.3)	232 (23.3)	
50th – 85th percentile	315 (39.8)	258 (26.0)	
> 95th percentile	78 (9.7)	409 (41.2)	
BMI (kg/m^2^)	17.5 (±3.1)	21.9 (±6.8)	***< 0.001***

Statistical significance set at *p*<0.05

## Discussion

In the past, national anthropometric pattern estimates in South Africa were incomplete; however a clearer anthropometric pattern per geographic area is slowly emerging. Overall there has been a consensus of a pattern of malnutrition and a recognition that higher obesity levels reside in more urbanized areas [2]. There is also a growing concern that these patterns of malnutrition and obesity are on the rise especially in children. Therefore, profiling these age groups are essential to highlight potential problems and direct future interventions. This is one of the largest South African studies to describe anthropometric profiles of 8–11 year old children in two distinct geographical areas. We present diverse patterns of malnutrition, both under- and over nutrition in two different geographical low-resource urban areas in South Africa.

This study found that the mean body mass index (BMI) of grade 4 learners from Johannesburg and Port Elizabeth was 19.9 kg/m^2^ (*n* = 1785), and that girls (*n* = 867) had a similar mean BMI (20.0 ± 5.8 kg/m^2^) compared to boys (19.9 ± 6.0 kg/m^2^) (*n* = 918). This is higher than a previous study done 12 years ago in South African primary school children [21], and yet is similar to the findings of the SANHANES conducted in 2012 [10]. The SANHANES showed that the mean BMI for the age group 10–14 years was 19.9 kg/m^2^ for girls (*n* = 691), and 18.0 kg/m^2^ for boys (*n* = 628). Previous data from South Africa as far back as 2004 showed secular trends of a decrease in stunting and a rise in overnutrition in children [22], and together with SANHANES data, our study indicates that BMI levels may have since continued to rise in this age group. Of great concern however, was that 32.1% of the population fell in the 50th- 85th percentile for BMI, while 27.3% fell in the 95th or greater percentile for BMI indicating that nearly half the population was either overweight or obese. This is well above prevalence rates of overweight (4–24.5%) and obesity (1–6.4%) as shown in a recent systematic review of South African children [23], as well as above national rates of 12.1% for overweight and 5% for obesity [23]. In contrast to the findings of the SANHANES survey where girls had a significantly higher prevalence rate than boys for overweight and obesity [10], it was found in this study that prevalence of overweight and obesity were fairly similar between girls and boys, where 32.8% of girls and 31.5% of boys fell within the 50th – 85th percentile BMI category and 27.5% of girls and 27.1% of boys fell within the 95th or greater percentile.

The South African population displays all the signs of being well established in the nutrition related non-communicable diseases phase of the nutrition transition continuum as determined by economic and social development, urbanization and acculturation [24–26]. While still faced with a double burden of under- and overnutrition, there is a definite shift in the prevalence of undernutrition and underweight, especially seen in children, towards an increase in the prevalence of overweight and obesity [22, 27]. Schools within low-resource communities are faced with many challenges when trying to promote healthy lifestyles for their learners, such as an absence of policies relating to healthy lifestyles, the regulation and availability of inexpensive, yet unhealthy food and snack foods from school tuck shops or street vendors outside school premises, as well as limited resources [28]. Contributing to the low physical activity levels of learners, is the fact that physical education as a stand-alone subject was phased out in 2004 to become one of four learning outcomes in ‘Life Orientation’ (LO) and the implementation of physical education therefore, becoming a challenge [28].

The findings from this study suggest that within South Africa different geographical areas are at different stages of the nutrition transition continuum, and this might be influenced by socio-demographic factors and economic development. Interesting data that emerged from our study was the anthropometric difference seen between the two geographical areas, Port Elizabeth and Johannesburg. Notable is the significant differences in BMI between the two areas, with more grade 4 learners in Port Elizabeth falling below the 15th percentile for BMI than Johannesburg (16.1% vs 9.5%), and on the other hand more learners in Johannesburg falling above the 95th percentile for BMI than in Port Elizabeth (41.2% vs 9.7%, *p* < 0.001), as indicated in Table 3. The national household travel survey (NHTS) 2013, reported that, in South Africa, 63,4% (Eastern Cape 75%) of learners walked all the way to school, with 5,5% (Eastern Cape 6,5%) of learners spending more than one hour a day walking to and from their schools [29]. The fact that more learners are walking to school in Port Elizabeth (Eastern Cape) than in other areas, e.g. Gauteng with 48% of learners reported walking to school [29], might be a contributing factor to the lower BMI seen in the Port Elizabeth cohort in our study.

Globalization is hastening the growth of South Africa’s largest cities into urban areas, but at different rates. According to Stats SA in 2011, the growth rate of Johannesburg was much more than that of Port Elizabeth (3.18% vs 1.36%), clearly indicating the urbanization is much more pronounced in Johannesburg. The possible resultant effect being on dietary intakes and lifestyle behaviours, such as women increasingly spending more time away from home, being at work, spending long hours travelling to and from work and therefore, relying more on convenience foods, high in fat and refined sugar, in-stead of preparing traditional low-fat, high-carbohydrate, high fibre diets at home to feed their families [9]. Previous studies showed that where mothers were working longer than 36 h per week, their children were at increased risk for being overweight or obese [9].

Unemployment rates are also much higher in Port Elizabeth than in Johannesburg (36.6% vs 25%) with more people per household (3.4) in Port Elizabeth than in Johannesburg (2.8). According to Mchiza et al. [30], households with fewer people, especially children, were positively associated with overweight or obese children. In addition, the unemployment rates in Port Elizabeth and the associated poverty may be a contributing factor towards the issue of food insecurity in the area. These issues of food insecurity, whereby adequate food is not accessible to households, may be demonstrated by the lower BMI shown in this area. Indeed, a previous South African study showed the prevalence of food insecurity was higher in the Eastern Cape (Port Elizabeth) than in Gauteng (Johannesburg), and more prevalent in rural versus urban areas [31]. According to the SANHANES the majority of women (64.5%) and 35.9% of men were influenced by the price of food and not health, when grocery shopping, as the cost of energy dense foods are less per unit than animal products or fruit and vegetables in South Africa [9].

The primary schools included in our study benefit from ‘The National School Nutrition Programme’ (NSNP), which was introduced in 1994 as a poverty alleviation strategy, by government, as part of the Reconstruction and Development Programme and aims to provide all learners in poorer primary and secondary schools (quintile 1,2 and 3 schools) with one nutritious meal being served per day. These meals consist of a protein (soya, fish, eggs, milk, beans or lentils), carbohydrate or starch (rice, bread, maize or samp), fats, oils and salt (added for taste) and a fruit or vegetable (served daily where possible) [32]. The NSNP goes a long way towards addressing under-nutrition and stunting in these low-resource communities, as well as various intervention programs run by NGO’s, in supporting schools to establish vegetable gardens and encouraging children to participate in physical activities and to eat healthy.

There are also differences in the levels of education between the two cities, with percentages of young adults (> 20 years old) having higher education being 12% in Port Elizabeth and 19.2% in Johannesburg, and those (> 20 years old) having completed secondary school at 30.5% in Port Elizabeth versus 34.7% in Johannesburg [31]. Education levels might also be a contributing factor in dietary and lifestyle behaviours, as higher levels of education may lead to more disposable income and an adoption of more western diets. Previous studies have shown that in developing countries, such as South Africa, the burden of NCD’s has shifted onto communities within the higher SES bracket and in employed people with better education and incomes, as opposed to high-income countries, where this burden is falling more on poor people with lower SES, more unemployment and less education [33].

This study contributes to the growing scientific evidence towards the increasing prevalence in overweight and obesity issues in South African children and the underlying problems of decreased physical activity and the consumption of energy-dense, but nutrient-poor foods, however the following limitations should be noted. Firstly, we used WHO BMI classifications for under- and overweight categories, although there has been much debate in the measurement and methods of classification in the paediatric population, making comparison between studies difficult [21, 23]. Furthermore, previous studies have shown differences in body composition between various ethnic populations [21, 22], whilst the current study concentrated on only gender and geographic differences. For the purposes of this study, we also excluded early adolescent learners from the analysis (12–14 year olds), although this age group may very well be especially vulnerable to increasing rates of overweight and obesity [34]. Despite these limitations, this study provides important cross-sectional data from two geographical areas that shows a transitional society and the commonly found issues of both under- and overnutrition.

## Conclusion

This study contributes critical formative data in describing the emerging problem of overweight and obesity observed in pre-pubertal children. Contrary to other South African data, our study showed little difference between boys and girls, however these difference may become more apparent with the onset of adolescence. We found that, overall, South African children appear to be at a higher risk of overnutrition than undernutrition. However, these patterns appear to be geographically specific. Urbanisation, and its resultant change in lifestyle behaviours, has left South African children living in urban areas at a high and increasing risk of overweight and obesity. Therefore, interventions to address malnutrition in South African, and possibly other LMICs undergoing similar nutrition transitions, should be area specific, with targeted intervention strategies aimed at this early childhood period being much needed.

## Abbreviations

BMI: Body Mass Index
LMICs: Low-to-middle income countries
WHO: World Health Organisation
